# Predictive value of microRNA let-7a expression for efficacy and prognosis of radiotherapy in patients with lung cancer brain metastasis: A case–control study

**DOI:** 10.1097/MD.0000000000012847

**Published:** 2018-11-02

**Authors:** Ji-Kuan Liu, Hong-Feng Liu, Yong Ding, Guo-Dong Gao

**Affiliations:** a Department of Thoracic Surgery, Jining No.1 People's Hospital, Jining; b Department of Surgery, Weishan People's Hospital, Weishan, Shandong Province, P.R. China.

**Keywords:** brain metastasis, DIECR1, efficacy, lung cancer, microRNAlet-7a, prognosis, radiotherapy

## Abstract

**Background:**

As a well-known cancer with high mortality, lung cancer has been implied to be closely associated with brain metastasis. Despite notable advances, effective treatment methods are still in urgent need. This study aims to investigate the value of serum microRNA-let-7a (miR-let-7a) expression in predicting efficacy and prognosis of radiotherapy in patients with lung cancer brain metastasis.

**Methods:**

To begin with, reverse transcription quantitative polymerase chain reaction (RT-qPCR) was performed for better understand of the correlation between miR-let-7a and lung cancer. Afterwards, the relationship between serum miR-let-7a expression and radiotherapy efficacy was analyzed by receiver operating characteristic curve analysis. Following successful transfection, RT-qPCR and Western blot assay were utilized for evaluating the involvement of miR-let-7a in regulation of DICER1 expression in lung cancer cell line. Then, whether miR-let-7a was implicated in proliferation and cell cycle distribution of lung cancer cells were confirmed by cell counting kit-8 assay and flow cytometry respectively.

**Results:**

Initially, it was revealed that serum miR-let-7a expression was decreased in lung cancer. Later, we found that decreased miR-let-7a displayed an unfavorable role in radiotherapy efficacy and overall survival rate of patients with lung cancer brain metastasis. After the successful transfection, the inverse relationship between miR-let-7a and DICER1 expression was uncovered. Meanwhile, biological behaviors of lung cancer cells were presented to be limited after transfection of overexpressed miR-let-7a.

**Conclusion:**

Our findings demonstrated that the lower expression of miR-let-7a in patients with lung cancer brain metastasis was closely related to unfavorable efficacy and prognosis of radiotherapy, and it may be an important predictive biomarker by regulation of DICER1.

## 1 Introduction

Lung cancer, one of the commonest shapes of primary lung tumors, has become the first greatest cause of death caused by cancer throughout the world, and is divided into 2 main types, small cell lung cancer (SCLC) and nonsmall-cell lung cancer (NSCLC).^[1–3]^ Although surgery is a potential therapeutic strategy in most primary tumors, surgical indication for patients with SCLC and NSCLC is usually marginal because of poor prognosis and local advancement, especially in subjects with metastases.^[4]^ Brain metastasis is the commonest neurological complication of systemic cancer; it happens in 20% to 40% of advanced malignancies patients and is closely correlated with poor prognosis and high morbidity in advanced lung cancer patients.^[5]^ Brain metastasis causes severe neurologic, emotional, and cognitive dysfunctions and its management and treatment depend on age, pathological state, the number of metastases at presentation, and status of systemic disease.^[6]^ Traditional treatments for brain metastasis includes radiotherapy, whole brain radiotherapy, stereotactic radiosurgery, and radiotherapy associated with surgery, but prognosis turns out to be poor with median survival of only 3 to 6 months in NSCLC and only 4 to 6 months in SCLC.^[7–9]^ Increasingly evidence indicate that microRNAs (miRs) is a target key involved in metastasis, and it could act as noninvasive biomarkers for combating brain metastasis.^[10,11]^

MiRs are a class of single-stranded noncoding RNAs with 19 to 25 nucleotides in length; they regulate gene expression by binding to complementary sequences in the 3′ untranslated region (UTR) of their target miRs, and participate in a variety of physiological and pathological processes.^[12,13]^ MiRs can also serve as oncogenes or tumor suppressor and thereby becoming useful biomarkers and therapeutic tools.^[14]^ As a prior report indicated, each microRNA-let-7a (miR-let-7a) family member involves in tumorigenesis, acting as a tumor suppressor.^[15]^ Previous study demonstrated thatmiR-let-7a could inhibit cell cycle progression in Ewing's sarcoma, a kind of disease with high degree of malignancy and rapidly metastasizing to lung and other tissues.^[16]^ Moreover, several researchers worldwide pointed out the downregulation of miR-let-7a in gastric cancer, pancreatic cancer and lung cancer.^[17–19]^ Shikeeva et al^[20]^ confirmed that miR-let-7a can be employed as a marker for poor prognosis of NSCLC and the low expression of miR-let-7a was related to the low expression of C/EBPα in patients with poor prognosis. It was also reported that in patients with NSCLC, the expression of miR-let-7a was significantly upregulated in the sensitive group as compared with that in patients with radiation resistance.^[21,22]^ Those reports all confirmed that miR-let-7a has a marker effect on the prognosis of NSCLC and is upregulated in radiosensitive patients, which is consistent with our research. However, the published literatures only found out the phenomenon, and the possible mechanism was not discussed. On the basis of this conclusion, a more longitudinal study has been conducted to confirm that the role of miR-let-7a as a prognostic marker for radiotherapy may be exerted by the regulation of DICER1. Thus, we conducted this study to investigate the relationship between serum miR-let-7a expression and radiotherapy efficacy in order to identify a potential biomarker capable of accurately predicting the efficacy and prognosis of radiotherapy in patients with lung cancer brain metastasis.

## 2 Subjects and methods

### 2.1 Ethics statement

The experimental protocols of the present study were approved by Ethics Committee of the Jining No.1 People's Hospital, and patients had a good understanding of this study and signed written informed consents in accordance with *Helsinki Declaratio*n.

### 2.2 Study subjects

From January 2012 to February 2015, 137 patients with lung cancer brain metastasis in Jining No.1 People's Hospital were selected as case group, including 82 males and 55 females. At the same time, 148 healthy individuals were recruited as control group, including 90 males and 58 females. Inclusion criteria: patients confirmed by pathological diagnosis, including clinical symptoms, physical signs and medical imaging detection, patients with no history of brain radiation therapy, patients aged between 41 and 70 years, patients with complete clinical and follow-up records, patients with positive attitudes toward treatment. Exclusive criteria: patients with radiotherapy intolerance, patients with documented history of brain disease.

### 2.3 Treatment methods

The patients in the case group were examined by magnetic resonance imaging (MRI) before and after 1 month of radiotherapy using a 3.0 T Philips Achieva MRI scanner with 8-channel phased array head coils. The detailed procedures were as follows: patients in the supine position received conventional contrast enhanced MRI of the head with Gd-DT-PA as the contrast agent (the dosage was 0.1 mol/kg; the injection flow rate was 2.0 mL/s). Then, the axial, coronal, and sagittal anatomy with 6 mm-thick section underwent MRI scans without interval. Treatment modality: a dose of 6 MV X-ray was administered to the patients in the supine position (a total of 40 Gy, divided into 20 times, 5 times/week).

### 2.4 Collection and preservation of serum samples

The venous blood of patients in the case group after radiotherapy and the healthy individuals in the control group were collected. After being drew, the blood was allowed to coagulate for 1 to 2 hours at 37°C (without anticoagulant). Then, the blood samples were placed in a refrigerator at 4°C overnight for clot retraction. After the serum separated naturally, the blood samples were centrifuged at the speed of 1610*g* for 10 minutes at 4°C. The serum was then collected and transferred to a clean tube, divided into small portions and stored in an ultralow temperature refrigerator at 4°C. During sample collection and preservation, the exposure time of samples should be as short as possible to prevent the degradation of RNA.

### 2.5 Reverse transcription quantitative polymerase chain reaction (RT-qPCR)

Total RNAs of tissues and cells was extracted from 200 μL of serum preserved in the ultralow temperature refrigerator using a miRNeasy Mini Kit (Qiagen GmbH, Hilden, Germany). RNA sample (5 μL) was diluted 20-fold with RNase-free ultrapure water (TIANGEN Biotechnology Co., Ltd, Beijing, China). The concentration and purity of RNA were determined with optical density (OD) value at 260 and 280 nm using an ultraviolet spectrophotometer. RNA samples with the OD_260_/OD_280_ ratio between 1.7 and 2.1 were considered as high purity to meet requirements of subsequent experiments. The cDNA template was synthesized by reverse transcription using PCR system. RT-qPCR was conducted using an ABI 7500 Real-Time PCR system (Applied Biosystems, Foster City, CA). The reaction system was 20 μL in volume, which consisted of 10 μL of 2× real time PCR buffer (Invitrogen, Carlsbad, CA) (containing 3 mmol/L Mg^2+^, 0.2 mmol/L dNTP and SYBR Green I fluorescent stains), 0.32 μL of forward and reverse specific primers (5 mmol/μL), 0.2 μL of Taq DNA polymerase (5 × 10^6^ U/L), 2 μL of cDNA template, and the remaining volume was replenished with RNase-free ultrapure water. The reaction conditions were as follows: predenaturation at 95°C for 3 minutes, followed by 30 cycles of denaturation at 94°C for 30 seconds, annealing at 55°C for 30 seconds, extension at 72°C for 30 seconds. The sequence of miR-let-7a forward primer was 5′-UGAGGUAGUAGGUUGUAUAGUU-3′, sequence of miR-let-7a reverse primer was 5′-CUAUACAACCUACUACCUCAUU-3′. The sequence of reference gene U6 forward primer was 5′-GAATTCCCCAGTGGAAAGACGC-3′, sequence of U6 reverse primer was 5′-GGTGTTTCGTCCTTTCCACAAGATATATAAAGGG-3′. The sequence of DICER1 forward primer was 5′-AAGGAAGCTGGCAAACAAGA-3′, sequence of DICER1 reverse primer was 5′-AAAACGAACCACCAAGTTGC-3′. The sequence of reference gene glyceraldehyde-3-phosphate dehydrogenase (GAPDH) forward primer was 5′-GAATTCCCCAGTGGAAAGACGC-3′, sequence of GAPDH reverse primer was 5′-CTCATGACCACAGTCCATGCCA-3′. Measurements for each sample were conducted in triplicate. PCR results were analyzed by the Opticon Monitor software version 3.0 (Bio-Rad Laboratories, Inc., Hercules, CA). The threshold was manually set at the lowest point of logarithmic amplification curve with parallel rise, and threshold cycle (Ct) values of each reaction tube were obtained. 2^−ΔΔCt^ method was employed for data analysis.^[23]^ The relative expression ratio of the targeting gene incase group and control group was expressed by 2^−ΔΔCt^, and the formula is as follows: ΔΔCt = (Ct_target gene_ − Ct_internal reference_) − Avg. (Ct_target gene_ − Ct_internal reference_). The experiment was conducted in triplicate.

### 2.6 Follow-up and efficacy evaluation of radiotherapy

Regular inspection and strict follow-up by telephone were carried out in the case group with detailed information of each patient recorded. Survival time was calculated from the date of diagnosis of brain metastasis to the death date or investigation end. Patients were followed up for 1 year with the investigation deadline was on February 29, 2016. No cases were censored.

According to response evaluation criteria in solid tumors (RECIST), complete response (CR), defined as disappearance of all target lesions; partial response (PR), defined as ≥30% reduction in the sum of the longest diameter from the baseline lesion; progressed disease (PD), defined as ≥20% raise in the sum of the longest diameter from the baseline lesion or appearance of new lesion; stable disease (SD), defined as other lesions except for CR, PR, and PD. CR + PR and SD + PD were grouped as sensitive and resistant groups respectively.

### 2.7 Cell culture and transfection

Lung cancer cell line A549 purchased from the cell bank of the Chinese Academy of Sciences (Shanghai, China) were cultured in Dulbecco's modified Eagle's medium containing 10% fetal bovine serum and double antibodies. When reached logarithmic growth phase, cells were inoculated into 25 mL culture flasks at a density of 1 × 10^5^ cells/flask, and incubated at 37°C in an incubator with 5% CO_2_ and 95% humidity.

Lipofectamine 2000 (Invitrogen) was used for transfecting miR-let-7a mimic in human lung cancer cell line A549 with PBS and let-7a-NC as controls. After 48 hours, the interference efficiency was measured for later molecular biology analysis.

### 2.8 Western blot assay

After transfection, the lung cancer cells were cultured until reached the logarithmic growth phase, and then the total protein was extracted as suggested by the protein extraction steps. The total protein concentration was measured by 2,2′-bicinchoninic acid (BCA) and the protein was transferred onto nitrocellulose membrane after subjected to sodium dodecyl sulfate–polyacrylamide gel electrophoresis (SDS–PAGE). Following mounting in 5% skimmed milk powder at room temperature for 1.5 hours, the membrane was washed with Tris-buffered saline Tween-20 (TBST), added with anti-DICER1 (1:1000) and β-actin (1:500) antibodies and incubated at 4°C overnight. After washing with TBST, horseradish peroxidase-labeled goat antirabbit secondary antibody (1:5000) was added into membrane for incubation at 37°C for 1 hour. After that, the membrane was washed with TBST again, and then developed via chemiluminescence. Images were acquired by ImageJ software with β-actin band as an internal reference and changes of DICER1 protein expression were observed. The experiment was repeated 3 times.

### 2.9 Cell counting kit (CCK)-8 assay

The cells in logarithmic growth phase were inoculated into a 96-well plate at a density of 5000 cells/well. In each group, 3 duplicated wells were set. At the same time, a blank medium without cells was set as a control. Cells were then cultured with 5% CO_2_ and 95% humidity at 37°C and after incubation for 24 hours, 10 μL of CCK-8 solution was added to the culture wells and incubated for another 2 hours. The OD value at 450 nm was measured with an American Biotek (Elx800) microplate reader. Average value of 3 times of OD value of each well minus OD value of the control was recorded.

### 2.10 Flow cytometry

After the density being adjusted to 5 × 10^4^ cells/mL, single cell suspension was seeded in a 6-well plate with 2 mL of culture medium per well added and cultured for 24 hours. Afterwards, the medium was replaced with a serum-free medium and the cells were cultured for 14 hours. Following that, cells were treated using a straw and counted, and then 5 × 10^5^/mL cells were centrifuged at 258*g* for 5 minutes with the supernatant discarded. For measuring apoptosis rate, firstly 500 μL of 1× binding buffer was added to resuspend the cells, followed by adding 5 μL of Annexin V-fluorescein isothiocyanate and 10 μL of 20 μg/mL propidium iodide (PI) successively. Then the above solutions were mixed and reacted under the environment void of light for 15 minutes. Flow cytometry was subsequently applied for analyzing the cell cycle and the steps were presented below: the cells to be detected were resuspended with 1 mL of precooled 75% alcohol at 4°C, fixed overnight at 4°C, rinsed with PBS, added with 150 μL of RNase inhibitor and 150 μL of 20 μg/mL PI, followed by reaction at room temperature for 30 minutes under the environment void of light. Finally, the flow cytometer was used to analyze the cell cycle.

### 2.11 Statistical analysis

The statistical analysis was conducted by SPSS 21.0 (IBM Corp., Armonk, NY). miR-let-7a expression was determined by 2^−ΔΔCt^ method, and patients were grouped into low expression group and high expression group based on the median of 2^−ΔΔCt^, which was taken as the critical value. The difference of miR-let-7a expression in patients with different therapeutic efficacies was analyzed by Pearson correlation analysis. The clinical value of serum miR-let-7a expression in efficacy prediction for patients with lung cancer brain metastasis was evaluated by receiver operating characteristic (ROC) curve. Univariate survival analysis was presented by Kaplan–Meier method and compared with log-rank test. Multivariate analysis of median overall survival (OS) was performed using the Cox proportional hazards regression model. *P* < .05 was deemed to statistically significant.

## 3 Results

### 3.1 Comparison of the baseline characteristics between the case group and the control group

Initially, we compared baseline features of study subjects between the case group and the control group. The average age of 137 patients in the case group was 60.88 ± 7.06 years, among which there were 82 males (59.85%). The average age of 148 health individuals in the control group was 59.97 ± 8.23 years, among which there were 90 males (60.81%). There was no statistic significant difference in age, gender, smoking history, and previous pulmonary diseases in the 2 groups (all *P* > .05) (Table 1).

**Table 1:**
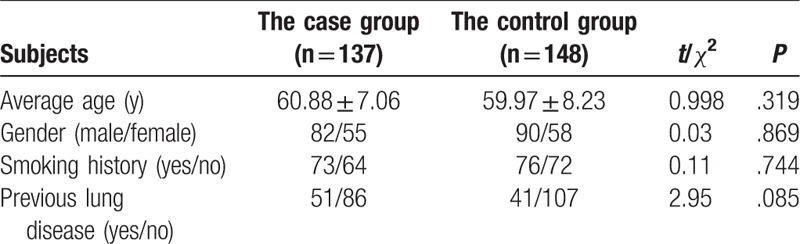
Comparison of baseline characteristics of lung cancer brain metastasis patients in the case group with healthy individuals in the control group.

### 3.2 Patients with lung cancer brain metastasis has downregulated miR-let-7a expression

**Figure 1:**
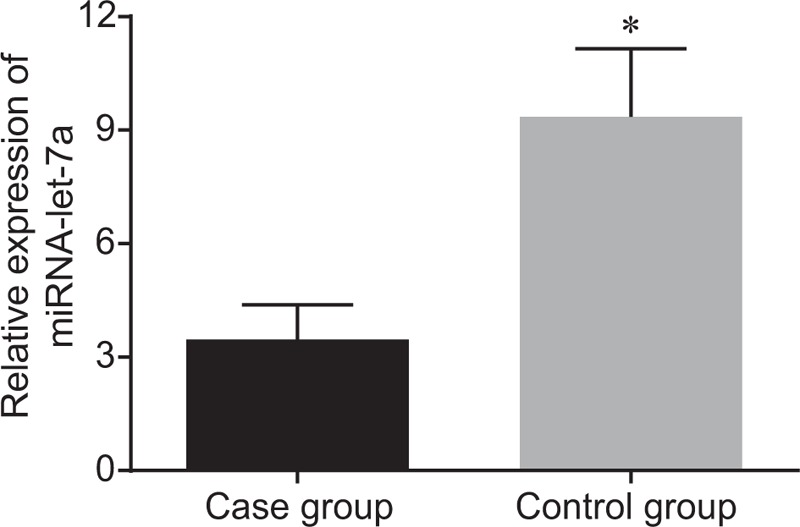
Serum miR-let-7a expression is downregulated inpatients with lung cancer brain metastasis. miR-let-7a = microRNA-let-7a.

Afterwards, RT-qPCR was applied for investigating miR-let-7a expression. The expression of miR-let-7a in serum in the control group was (9.35 ± 1.81), while the expression of miR-let-7a in serum in the case group was (3.46 ± 0.92). Serum miR-let-7a expression was significantly downregulated in patients with lung cancer brain metastasis (*P* < .05) (Fig. 1). Taken together, lung cancer brain metastasis patients presented decreasedmiR-let-7a expression in serum.

### 3.3 Correlation between the relative expression of miR-let-7a and clinicopathologic features of patients with lung cancer brain metastasis

Next, clinicopathologic features of patients with lung cancer brain metastasis in the case group were in comparison. In the initial survey, serum miR-let-7a expression in serum of patients varied with Karnofsky Performance Status (KPS), pathological type, and tumor nodes metastasis (TNM) stage. The relative expression of miR-let-7a in serum was lower in patients with KPS ≤ 70, NSCLC or III + IV stage compared with that in those patients with KPS > 70, SCLC or I + II stage (*P* < .05). There was no statistic difference of serum miR-let-7a expression in patients characterized by other clinical features (age, smoking, gender, history of pulmonary disease) (*P* > .05) (Table 2).

**Table 2:**
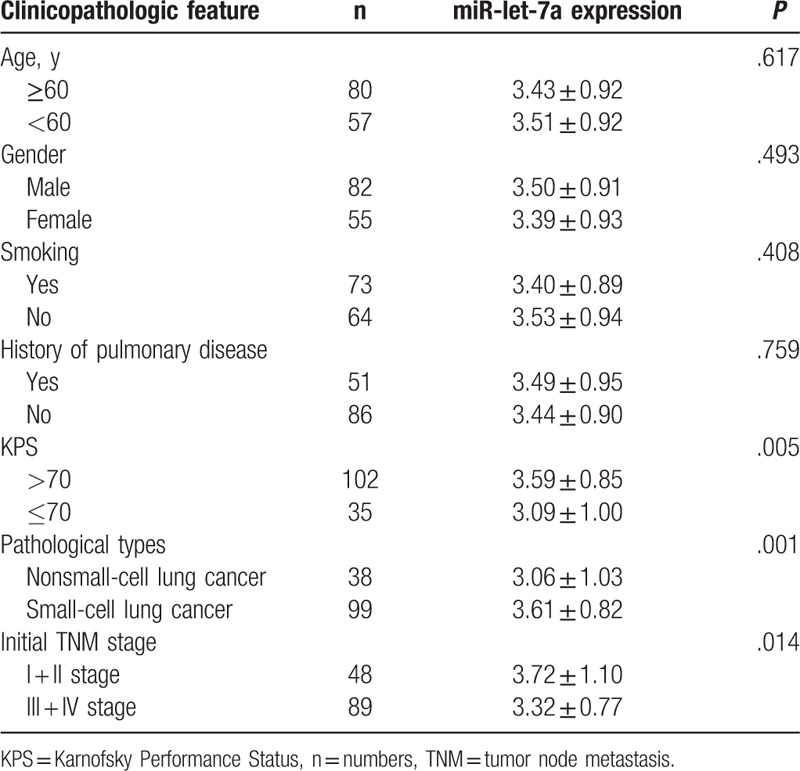
Correlation between serum miR-let-7a expression and clinicopathologic features of patients with lung cancer brain metastasis in the case group.

### 3.4 Predictive efficiency of serum miR-let-7a expression has a positive relationship with radiotherapy efficacy on patients with lung cancer brain metastasis

**Figure 2:**
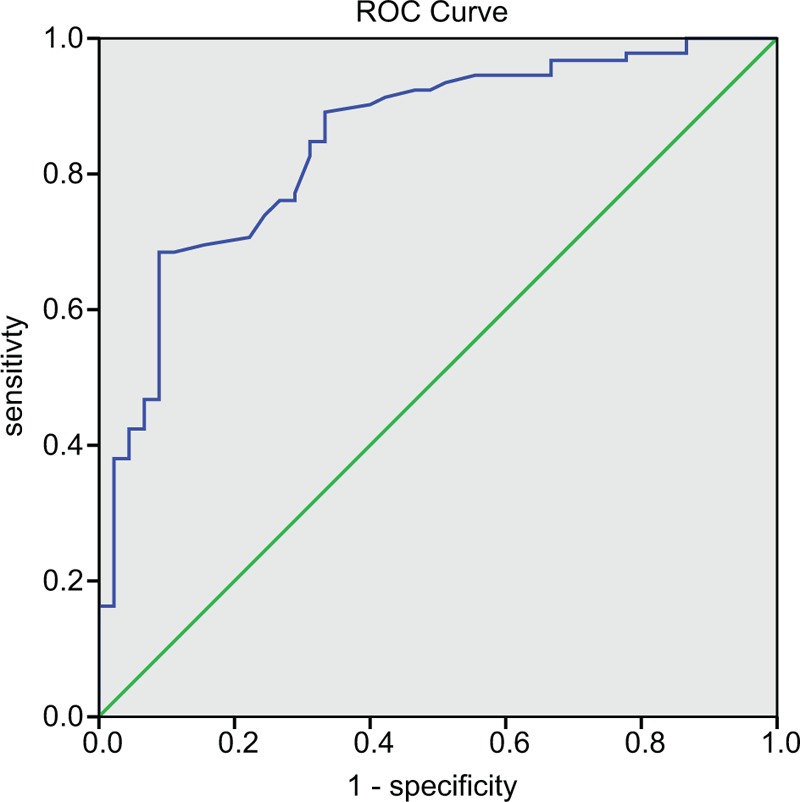
Predictive efficiency of serum miR-let-7a expression positively impacts radiotherapy efficacy of patients in the case group. ROC = receiver operating characteristic.

Later, predictive value of serum miR-let-7a expression in radiotherapy efficacy (sensitive or resistant) was analyzed based on ROC analysis. Area under the curve (AUC) was 0.847, and the optimal threshold was 3.34. The sensitivity and specificity of serum miR-let-7a expression in predicting radiotherapy efficacy were 68.5% and 91.1%, respectively. These results indicated that serum miR-let-7a expression had diagnostic and predictive value for radiotherapy efficacy (Fig. 2). In conclusion, miR-let-7a expression was positively associated with radiotherapy efficacy.

### 3.5 Higher miR-let-7a expression improves radiotherapy efficacy on patients with lung cancer brain metastasis

Then, ROC analysis was again utilized for measuring the relationship between miR-let-7a expression and radiotherapy efficacy. Subjects in case group were classified as patients with high expression (miR-let-7a level ≥ 3.34) and patients with low expression (miR-let-7a level < 3.34). Low expression rate of miR-let-7 in the sensitive group was 31.52%, which was significantly lower than that in the resistant group (91.11%) (*P* < .05). The result is exhibited in Table 3. Taken together, miR-let-7a was a positive factor for radiotherapy efficacy of patients with lung cancer brain metastasis.

**Table 3:**
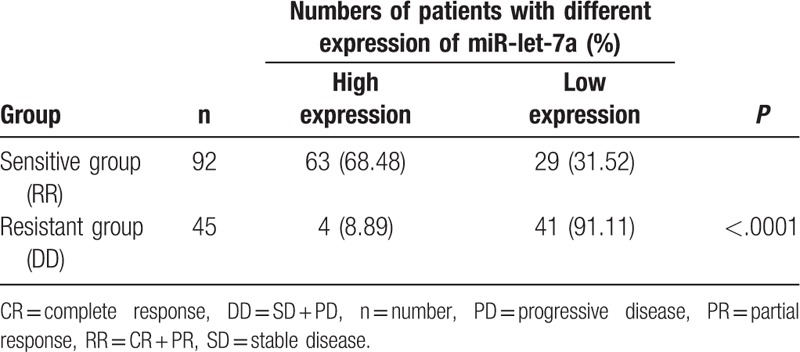
The expression of miR-let-7a in sensitive group and resistant group.

### 3.6 MiR-let-7a expression prolongs OS of patients with lung cancer brain metastasis after radiotherapy

**Figure 3:**
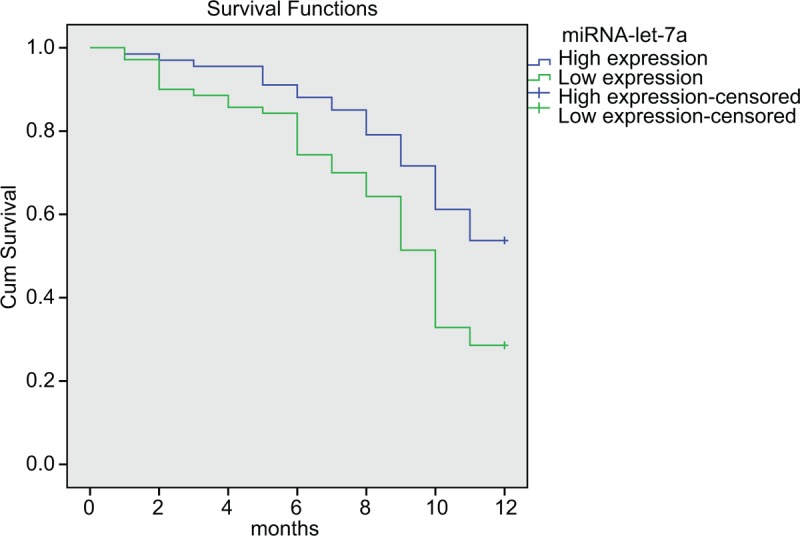
MiR-let-7a positively effects on radiotherapy efficacy of patients in the case group. miR-let-7a = microRNA-let-7a.

In the following experiment, we determined the effect of miR-let-7a on OS of lung cancer patients after radiotherapy. Up to the end of the follow-up, median OS of 137 patients were 10 months, and 1-year OS rate was 40.88%. The results of univariate survival analysis showed that KPS, pathological type, TNM stage and serum miR-let-7a expression were closely related to OS (*P* < .05) (Table 4). The 4 factors (KPS, pathological type, TNM stage and miR-let-7a expression) were included in the Cox proportional hazard model, and the analysis results showed that the KPS, TNM stage and miR-let-7a expression were independent prognostic factors in patients with lung cancer brain metastasis after radiotherapy (all *P* < .05) (Table 5, Fig. 3).

**Table 4:**
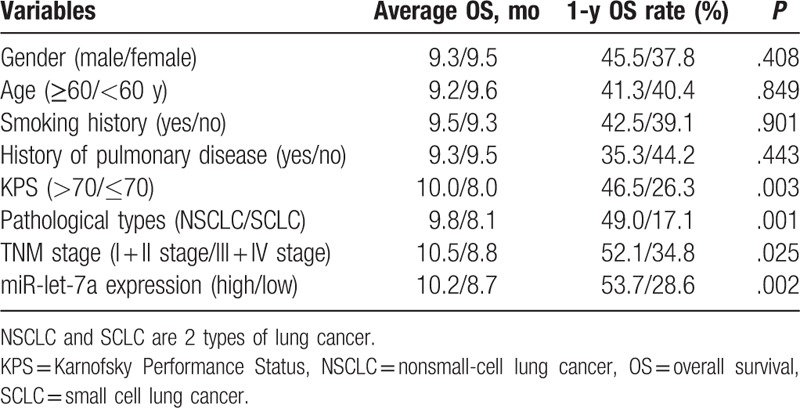
Univariate survival analysis of OS in patients with lung cancer brain metastasis.

**Table 5:**
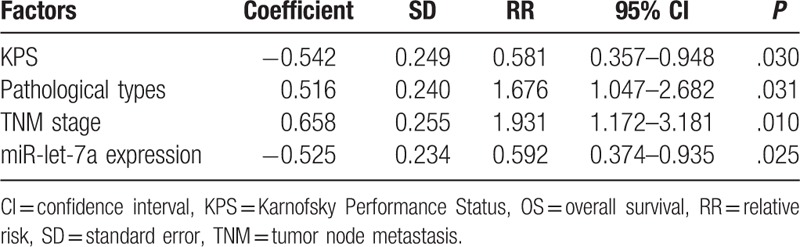
Multivariate Cox regression analysis of OS in patients with lung cancer brain metastasis.

### 3.7 Successful establishment of lung cancer cell line with high expression of miR-let-7a

**Figure 4:**
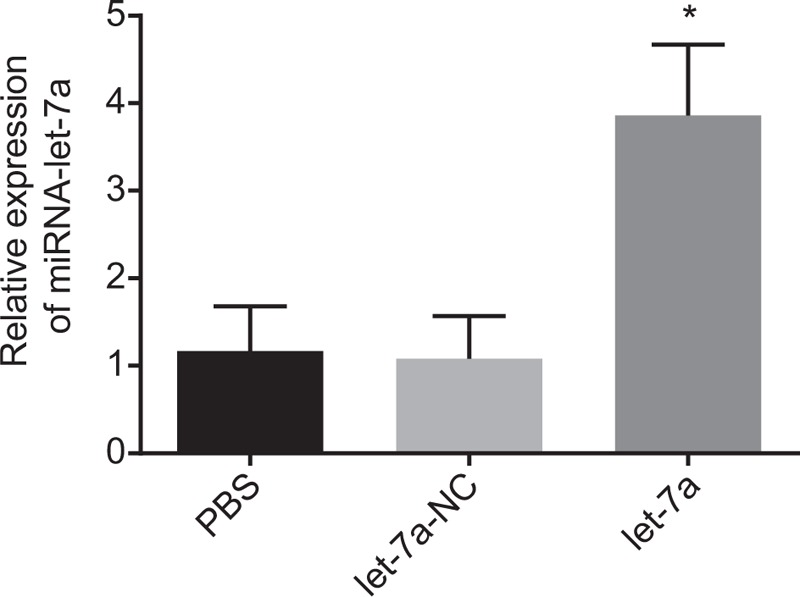
Successful construction of cell line with abnormal expression of miR-let-7a. ^∗^*P* < .05 vs cells transfected with PBS or NC. miR-let-7a = microRNA-let-7a.

Subsequently, whether lung cancer cell line was successfully constructed was explored. After transfection of miRNA let-7a mimic in lung cancer cell line A549, the expression of miRNA let-7a was significantly increased compared to cell line transfected with control (Fig. 4) (*P* < .05). This indicated that the miR-let-7a lung cancer cell line was successfully constructed.

### 3.8 MiR-let-7a downregulates DIECR1 expression

**Figure 5:**
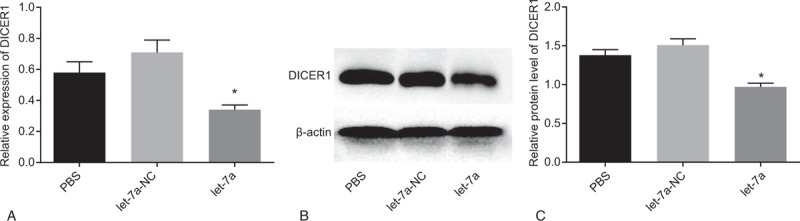
MiR-let-7a lowers expression of DIECR1. (A) mRNA levels of DIECR1 in response to the treatment of PBS, let-7a-NC, and let-7a; (B) the gray value analysis of DIECR1 protein band in response to the treatment of PBS, let-7a-NC, and let-7a; (C) protein levels of DIECR1 in response to the treatment of PBS, let-7a-NC, and let-7a; ^∗^*P* < .05 vs cells transfected with PBS or NC; miR-let-7a = microRNA-let-7a.

In addition, the correlation between miR-let-7a and DIECRI was investigated. As suggested in Fig. 5, transfection of miRNA let-7a mimic resulted in lower expression of mRNA and protein expression of DIECRI (*P* < .05). Taken this result into consideration, we estimated that miR-let-7a expression had an inverse relationship with DIECRI expression.

### 3.9 MiR-let-7a delays proliferation of lung cancer cells

**Figure 6:**
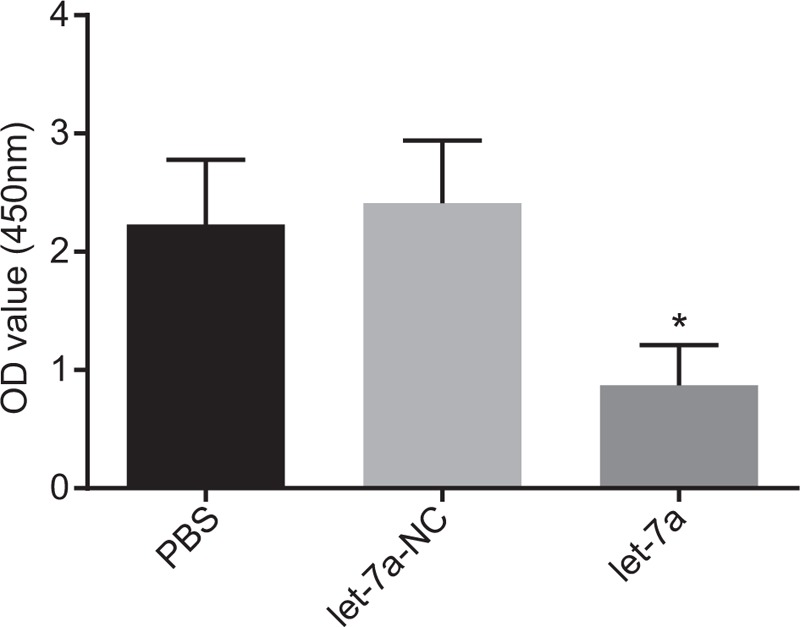
MiR-let-7a exerts a negative effect on proliferation of lung cancer cells. ^∗^*P* < .05 vs cells transfected with PBS or NC; miR-let-7a = microRNA-let-7a.

**Figure 7:**
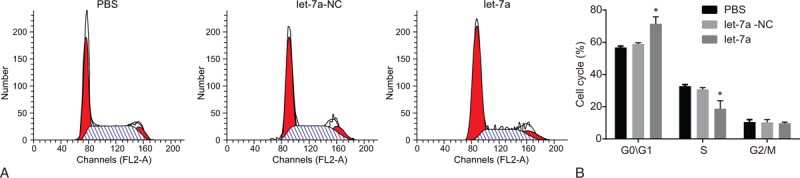
MiR-let-7a blocks cell cycle. (A) Flow cytometry map demonstrating cell cycles; (B) flow cytometry results validated that miR-let-7a prolongs G0/G1 phase while shortening S phase; ^∗^*P* < .05 vs cells transfected with PBS or NC; miR-let-7a = microRNA-let-7a.

Finally, CCK-8 was conducted to confirm the involvement of miR-let-7a in lung cancer cell proliferation. After transfection of miRNA let-7a mimic, lung cancer cell line A549 had reduced cell proliferation compared with the cells transfected with PBS and miR-let-7a-NC (*P* < .05) (Fig. 6), which suggested that miRNA let-7a was a negative factor for lung cancer proliferation. Compared with the cells transfected with PBS and miR-let-7a-NC, more cells were arrested in the G0/G1 phase and less in the S phase in cells with transfection of miR-let-7a (*P* < .05) (Fig. 7). In conclusion, it was suggested that miR-let-7a conferred resistance to proliferation of lung cancer cells.

## 4 Discussion

Brain metastasis, which most frequently occurs in lung cancer and breast cancer with extra high morbidity and mortality, is common and fatal once formed in cancer patients.^[24,25]^ Approximately 20% to 40% NSCLC patients will develop brain metastases, and the main treatments include whole brain radiotherapy, stereotactic radiosurgery, and surgical resection.^[26]^ In our study, we explored the relationship between serum miR-let-7a expression and radiotherapy efficacy in patients with lung cancer brain metastasis. We found that serum miR-let-7a expression in patients with lung cancer brain metastasis was closely related to the efficacy and prognosis of radiotherapy, and it could be an important predictive biomarker.

Our study presented that miR-let-7a expression in serum in the case group was significantly lower than that in the control group, and downregulation of miR-let-7a expression implied poor outcomes. Abnormal expression of miRs is regarded as an intrinsic characteristic of cancer growth and development, which indicated the clinical potential of miRs as biomarkers for diagnosis, prognosis, and prediction of curative outcomes.^[27]^ Consistent with our finding, several researches worldwide proposed that let-7 expression was observed at a low level in human lung cancers.^[28–30]^ Besides that, a relevant research presented that miR-let-7 expression was remarkably decreased in tumor-initiating cells of breast cancer and raised with cell differentiation, and on the contrary, miR-let-7 expression was shown to reduce cell proliferation as well as in vivo tumor formation and metastasis.^[31]^ Additionally, it was also reported that inhibiting miR-let-7 could increase protein content of transcription factor BACH1 and its targets HMGA2 and MMP1, all of which were known to promote bone metastasis.^[32]^ Our study also revealed that DIECR1 expressed at a low level and was downregulated by miR-let-7a. Similarly, a recent study pointed out that declined DIECR expression was observed in oral cancer.^[33]^ In line with our results, the finding that miR-let-7a downregulated DIECR1 expression has been supported by a previous study.^[34]^ Moreover, the negative correlation between miR-let-7a and DIECR has been investigated and proposed previously.^[33,35,36]^ And then, as reflected in our study, serum miR-let-7a expression in serum of patients varied with KPS, pathological type, and TNM stage, which made serum miR-let-7a a larvaceous biomarker for early diagnose of the disease, risk assessment, and prognosis. According to the existing research, low miR-let-7a expression was significantly correlated with low-moderate differentiation, lymph node metastasis N1–N3 and stage III–IV, indicating that low miR-let-7a expression could be an independent factor for the prognosis of gastric cancer.^[17]^ Besides, low miR-let-7a-2 expression was related to poor survival in lung adenocarcinoma patients, suggesting that low miR-let-7a expression acted an independent risk factor for prognosis of lung cancer.^[37]^ In addition, our results showed that low expression rate of miR-let-7a was significantly declined in sensitive group when compared with the resistant group after radiotherapy, which proved that miR-let-7a had diagnostic and predicative value for radiotherapy efficacy. As is known to all, miRs could be regarded as new noninvasive biomarkers for cancer diagnosis.^[38]^ The experimental data of other study suggested that miR-let-7a had diagnostic and predicative value in patients with myelodysplasia and high miR-let-7a expression was independent predictive factor for OS.^[39,40]^ Besides, one study before indicated that miR-let-7 family could enhance sensibility to radiation in lung cancer cells, and downregulation of miR-let-7 expression in NSCLC would increase risk of postoperative death after surgical treatment.^[37]^

Subsequently, we found that reduced cell proliferation was caused after transfection of miR-let-7a. Meanwhile, cell cycle distribution pattern was altered with more cells in G0/G1 phase and less in S phase. A previous report focusing on lung cancer mentioned that let-7 mRNA family acted as an inhibitor in cellular proliferation.^[31]^ In line with this study finding, several studies previously demonstrated that miR-let-7a was a tumor-suppressing gene in different human cancers, such as prostate cancer, laryngeal squamous cancer and endometrial carcinoma, and it was related to enhanced cancer aggressiveness and adverse clinical outcome.^[41–45]^ Correspondingly, other studies found that overexpression of miR-let-7a was able to inhibit the proliferation, migration and invasion in lung cancer and gastric cancer by repressing cellular proliferation pathways.^[17,19]^ In addition, miR-let-7a was able to delay the tumor development of lung cancer transplanted subcutaneously in nude mice by regulating k-Ras and c-Myc expression, which indicated its role as a perspective curative target for lung cancer therapy.^[46]^ Except miR-let-7a, more and more other members of let-7 mRNA family have received attention due to their function of delaying progression of diverse cancers. For instance, miR let-7g and let-7i were responsible of inhibiting proliferation while inducing apoptosis of human esophageal carcinoma cells via interaction with the drug transporter ABCC10.^[47]^ What is more, by targeting the proto-oncogene N-myc proto-oncogene protein, miR let-7e was found to negatively influence cell proliferation in MYCN-amplified neuroblastoma.^[48]^

In summary, we provided strong and sound evidence that low miR-let-7a expression in serum samples of patients with lung cancer brain metastasis was related to poor prognosis and it could effectively predict radiotherapy efficacy. There is a limitation in our study, the results showed that 30% of the 92 patients in the sensitive group had low expression of miR-let-7a. The explanation may result from 2 reasons, first, the sample size is small and cannot represent the large sample population. The sample size may affect the definition of the boundary value. Second, there were variations in the process of sampling and processing of different subjects, which increased the variation of miR-let-7a expression. Later, we will verify the above conclusions in experiments with a larger sample size, and standardize the sampling and processing process to reduce bias.

## Acknowledgment

We would like to acknowledge the helpful comments on this paper received from our reviewers.

## Author contributions

**Conceptualization:** Ji-Kuan Liu, Hong-Feng Liu, Yong Ding, Guo-Dong Gao.

**Data curation:** Ji-Kuan Liu, Yong Ding, Guo-Dong Gao.

**Formal analysis:** Hong-Feng Liu, Yong Ding, Guo-Dong Gao.

**Investigation:** Hong-Feng Liu, Guo-Dong Gao.

**Methodology:** Hong-Feng Liu, Yong Ding, Guo-Dong Gao.

**Project administration:** Hong-Feng Liu.

**Resources:** Hong-Feng Liu, Yong Ding, Guo-Dong Gao.

**Software:** Hong-Feng Liu.

**Supervision:** Ji-Kuan Liu, Hong-Feng Liu, Yong Ding, Guo-Dong Gao.

**Validation:** Ji-Kuan Liu, Hong-Feng Liu, Yong Ding.

**Visualization:** Hong-Feng Liu, Yong Ding, Guo-Dong Gao.

**Writing – original draft:** Ji-Kuan Liu, Hong-Feng Liu, Yong Ding.

**Writing – review & editing:** Ji-Kuan Liu, Hong-Feng Liu, Yong Ding, Guo-Dong Gao.
